# Regulatory role of the programmed cell death 1 signaling pathway in sepsis induced immunosuppression

**DOI:** 10.3389/fimmu.2023.1183542

**Published:** 2023-05-24

**Authors:** Shubai Zhong, Yuanqin Yin

**Affiliations:** ^1^Department of Critical Care Medicine, Shengjing Hospital of China Medical University, Shenyang, Liaoning, China; ^2^Cancer Institute, First Affiliated Hospital, China Medical University, Shenyang, Liaoning, China

**Keywords:** sepsis, programmed cell death 1, immunosuppression, immunomodulation, immunotherapy

## Abstract

Sepsis is a multiple organ dysfunction syndrome caused by the host’s immune response to infection, with extremely high incidence and mortality. Immunosuppression is an essential pathophysiological alteration that influences the clinical treatment and prognosis of sepsis. Recent studies have suggested that the programmed cell death 1 signaling pathway is involved in the formation of immunosuppression in sepsis. In this review, we systematically present the mechanisms of immune dysregulation in sepsis and elucidate the expression and regulatory effects of the programmed cell death 1 signaling pathway on immune cells associated with sepsis. We then specify current research developments and prospects for the application of the programmed cell death 1 signaling pathway in immunomodulatory therapy for sepsis. Several open questions and future research are discussed at the end.

## 1 Introduction

Sepsis is the most serious threat to human health worldwide, affecting more than 40 million people worldwide each year and killing roughly one in five of them (1). Its core mechanism is the host immune response disorder caused by infection, which leads to multiple organ dysfunction (2). Current studies believe that immunosuppression originates from the very early stage of sepsis, which is caused by the excessive release of anti-inflammatory cytokines and the decline in the number and function of immune cells, and continuous immunosuppression will lead to increased mortality (3). Programmed cell death 1 (PD-1) signaling pathway plays an crucial role in immune regulation of tumor and autoimmune diseases (4). It has recently been reported that the PD-1 pathway is also involved in the formation of immunosuppression in sepsis, and blocking this pathway has been shown to have promising therapeutic effects in animal experiments. Therefore, the purpose of this article is to introduce the mechanism of immune dysregulation in sepsis, demonstrate the regulatory effects of PD-1 signaling pathway on various kinds of sepsis-related immune cells, and explore the application prospect of blocking PD-1 signaling pathway in immunomodulatory therapy of sepsis and problems to be solved in future research. All references cited in this paper are from the PubMed database.

## 2 Immune dysregulation in sepsis

The pathogenesis of sepsis is the immune disorder caused by infection, which includes the excessive release of pro-inflammatory cytokines and the immunosuppression, both of which occur simultaneously at the early stage of infection (5). With the help of antibiotics and infection control measures, the body will return to homeostasis if the host’s immune system is able to rid itself of the pathogen quickly. However, some hosts showed continuous immunosuppression, whose immune system could not clear the pathogens in time, and even developed to secondary infections, eventually into multiple organ dysfunction syndrome (MODS) and death (6).

In the early stage of sepsis, the body’s innate immune system first recognizes the pathogen-associated molecular patterns (PAMPs) after the pathogen has invaded, causing the release of a series of pro-inflammatory cytokines such as tumor necrosis factor-alpha (TNF-α), interleukin-1 (IL-1) and interleukin-6 (IL-6). These pro-inflammatory factors promote downstream cytokine release, causing tissue damage, disseminated intravascular coagulation (DIC), and organ dysfunction (7), also called a cytokines storm. At the same time, immune cells secrete anti-inflammatory cytokines like interleukin-4 (IL-4) and interleukin-10 (IL-10). Secreted mainly by activated T lymphocytes, IL-4 can induce CD4+ T cells converting into T helper 2 (Th2) cells, and indirectly inhibit the release of interleukin-2 (IL-2), interferon-gamma (IFN-γ) and other pro-inflammatory factors (8). IL-10 is mainly secreted by monocytes and macrophages, and can promote the proliferation of immunosuppressive cells like regulatory T cells (Tregs) and myeloid-derived suppressor cells (MDSCs) (9). Thus, the negative regulatory effect of anti-inflammatory cytokines is a major cause of immunosuppression in sepsis.

In addition, multiple factors contribute to the reduction in the number and function of immune cells associated with sepsis. In patients with sepsis, over-expressed caspase-1 or caspase-4/5 combined with lipopolysaccharide (LPS) recognizes and activates gasdermin-D (GSDMD), an actor of pyroptosis, which can cause immune cell death (10). Patients with sepsis showed significantly reduced expression of human leukocyte antigen-DR (HLA-DR), which resulted in decreased antigen presenting cell function, and further inhibited subsequent T cell activation and adaptive immune response (11). Negative costimulatory molecules, such as PD-1, T-cell immunoglobulin and mucin domain-containing protein-3 (TIM-3) and B and T lymphocyte attenuator (BTLA) is also highly expressed in sepsis, leading to immunosuppression (12). The precise role of PD-1 will be discussed later.

## 3 Expression of PD-1 pathway related molecules in sepsis

PD-1, a member of the B7-CD28 immunoglobulin superfamily, is a transmembrane receptor protein that acts as an essential negative costimulatory molecule, also known as an immune checkpoint, and is widely expressed on the surfaces of lymphocytes, dendritic cells, monocytes and other immune cells. After binding with Programmed death-ligand 1(PD-L1), PD-1 inhibits proliferation and cytokins secretion of T cells by dephosphorylation of the T cell receptors (TCR) related molecules, and promotes the conversion of T cells to Tregs, thus transmits negative immune regulatory signals (13). Since PD-L1 is widely expressed on tumor cells, immunosuppression mediated by PD-1 pathway is an critical mechanism for immune escape of tumor cells (14).

Similar to tumor cells, PD-1 and its ligands are also highly expressed in various immune cells associated with sepsis. The expression of PD-1 in spleen T cells and PD-L1 in macrophages increases in candidiemic mice (15).CD4+ T cells in septic mice showed a special depletion phenotype, which was characterized by increased expression of PD-1, TIM-3 and the proportion of Tregs (16). PD-L1 expression also increases in Myeloid derived suppressor cells (MDSCs) in septic mice (17). Human immune cells also highly express PD-1 related molecules during sepsis. PD-1 expression increases in CD4+T cells in patients with sepsis and is associated with a poor prognosis. The cutoff value for predicting death in 28 days with the expression frequency of PD-1 was 22.46% (18). Expression of immune checkpoints including PD-1 by peripheral blood mononuclear cells (PBMCs) increased in patients with aggressive candida infection, especially in patients who died (19). The expression of PD-L1 in natural killer (NK) cells in septic patients is significantly increased, and its frequency is an independent risk factor for death at 28 days (20). Soluble PD-L1(sPD-L1) in circulation is still increased even after recovery from sepsis, with significantly higher readmissions and all-cause mortality within six months (21). PD-1/PD-L1 expression was even increased in various subsets of memory B cells and T cells in patients with sepsis (22). These findings confirm that PD-1 pathway related molecules are widely expressed in immune cells associated with sepsis.

## 4 Regulatory effects of the PD-1 pathway on immune cells associated with sepsis

It has been established that the PD-1 signaling pathway, based on extensive expression, plays a regulatory role in various types of innate and adaptive immune cells associated with sepsis.

### 4.1 Regulatory effects on neutrophils

PD-L1 expression was negatively correlated with the apoptosis rate of neutrophils in septic patients by prolongating neutrophil survival through activation of PI3K/Akt signaling pathway (23). PD-L1 expression also reduced the migration ability of neutrophils and induced apoptosis of co-cultured lymphocytes in septic mice (24).

### 4.2 Regulatory effects on monocytes/macrophages

In septic mice with PD-1 gene deletion, Kupffer cells expressed more major histocompatibility complex II (MHC-II), engulf function was significantly enhanced, and after LPS stimulation, the secretion of IL-6 and monocyte chemoattractant protein-1 also increased significantly. In addition, PD-1 gene deletion was found to decrease cleaved caspase-3 levels and reduce LPS induced Kupffer cell apoptosis (25). In septic mice, blocking PD-1 with antibodies resulted in aggregation of macrophage cytoskeleton proteins α-actitinin and F-actin, enhancing their phagocytosis and mobility. It is speculated that the PD-1 signaling pathway achieves this effect by inducing cytoskeletal rearrangement (26). Avendano-Ortiz et al. found that the expression of PD-1 in monocytes of septic patients is related to endotoxin tolerance, and blocking this pathway can enhance the immune response of monocytes in sepsis patients (27).

### 4.3 Regulatory effects on dendritic cells

In septic mice, TNF-α induced protein 8 like 1 (TIPE1) can inhibit dendritic cell maturation by activating PD-1 signaling pathway, thus affecting its antigen presentation ability and subsequent T cell activation (28).

### 4.4 Regulatory effects on adaptive immune cells

Besides the typical effects of inducing T-cell exhaustion and promoting the transformation from efficient T cells to Tregs that mentioned previously (13), PD-1 pathway can also inhibit the cytokines secretion of T cells. The ability of CD4+T cells to secrete TNF-α was decreased in septic mice with elevated PD-1 expression (29). Similar phenomena have also been observed from the *in vitro* experiments of septic patients, and PD-1 blockers can enhance the ability of T cells to secrete IFN-γ (30, 31). In addition, the PD-1 pathway can also inhibit activity and secretion ability of CD19(+) B cells (32), but its specificity in sepsis is still lacking.

## 5 Blocking the PD-1 pathway in immunomodulatory therapy for sepsis

It was previously believed that the main harm of sepsis was systemic inflammatory response syndrome (SIRS) caused by infection, which was induced by excessive release of inflammatory cytokines in the early stage of the disease. Therefore, the immunotherapeutic strategies of sepsis in the past were mainly to inhibit the inflammatory response and block the pro-inflammatory cytokines such as PAMPs, Toll-like cytokines (TLRs), TNF-α, IL-6, etc., but the results were not as expected. Although the fatality rate at the early stage of sepsis decreased, the overall prognosis did not continue to improve, and more patients showed immunosuppression, uncontrolled infection and organ failure in the late stage of the disease (3, 33, 34). This led to a deeper understanding of the core mechanism of sepsis, the now widely accepted theory of immune dysregulation. It also provides a theoretical basis for animal and clinical trials of immunomodulatory therapies for sepsis, which attenuate immunosuppression by blocking the PD-1 pathway, as shown in **Table 1**.

**Table 1:** Studies on blocking the PD-1 pathway in sepsis.

Study model	Intervention against the PD-1 pathway	Result	Reference
Septic mice	PD-1 knockout	Increased aggregation of neutrophils in the abdomen, increased levels of inflammatory factors in the blood, and improved overall survival	Young WA, et al., 2017 (35)
	Anti-PD-1 antibodies	Improved the bacterial clearance and survival rate	Patil NK, et al., 2018 (36)
	Peptide-based PD-1 inhibitors	Enhanced macrophage function, pathogen clearance and survival	Kotraiah V, et al., 2020 (37)
	Restored the expression of PD-L1 gene by adenovirus transduction	Reduced liver damage and improved survival	von Knethen et al., 2019 (38)
Septic patients	Nivolumab, a monoclonal antibody of PD-1	Increased HLA-DR expression and decreased IL-6 levels in monocytes; No safety accidents discovery	Hotchkiss RS, et al., 2019 (39)

PD-1, Programmed cell death 1; PD-L1, Programmed cell death ligand 1; HLA-DR, human leukocyte antigen-DR; IL-6, interleukin-6.

### 5.1 Animal trials

Multiple animal trials have demonstrated that blocking the PD-1 pathway or knockout of related genes can improve the prognosis of sepsis. Compared with wild-type, PD-1 knockout mice showed increased aggregation of neutrophils in the abdomen after sepsis, increased levels of inflammatory factors in the blood, and significantly improved overall survival (35). After receiving PD-L1 antibody treatment, septic mice caused by burns showed an improvement in clearance of pathogenic bacteria and survival rate (36). Treatment of septic mice with peptide-based PD-1 inhibitors enhanced their macrophage function, pathogen clearance, and survival (37). However, not all animal trials have reached similar conclusions. One study found that PD-L1 expression was down-regulated in liver cells of septic mice. But after the expression of PD-L1 gene was restored by adenovirus transduction, the damage of liver cells in mice was reduced and the survival rate was significantly improved (38).Possible reasons for this discrepancy will be discussed later.

### 5.2 Clinical trials

Clinical trials of therapies that block the PD-1 pathway are in their infancy compared to animal trials. Hotchkiss et al. (39) selected 31 septic patients from the Intensive care unit (ICU) of 10 hospitals in the United States and conducted a randomized controlled trial on nivolumab, a monoclonal antibody of PD-1, at different doses. Basic pharmacokinetic parameters were obtained and increased HLA-DR expression and decreased IL-6 levels were observed in monocytes in all patients. But the study was a Phase 1 trial that demonstrated the safety of nivolumab only in patients with sepsis, not its efficacy. Another randomized controlled clinical trial of anti-PD-L1 drugs and placebo conducted by the same team also reached a similar conclusion (40). No follow-up clinical trials were reported at the time of submission.

## 6 Problems and prospects

As previously mentioned, PD-1/PD-L1 plays an essential regulatory role in the immunosuppression induced by sepsis by affecting the function of various immune cells and is closely related to the immune status and prognosis of sepsis. Most animal studies have shown that blocking the PD-1 pathway improves survival, and preliminary clinical studies have demonstrated the safety of related drugs in humans. However, a variety of fundamental and clinical issues remain to be addressed in this field.

### 6.1 Role of PD-L2

PD-L2 is another ligand of PD-1, which is mainly expressed on the surface of macrophages and dendritic cells and has a stronger affinity for PD-1, with particular expression in some tumor cells (41). In septic mice with PD-L2 gene deficiency, hepatic vascular permeability increased and liver damage was more severe, but inflammatory factors such as IL-6 decreased instead, and there was no difference in survival rate overall (42). Currently, research on PD-L2 is extremely limited and its regulatory role in sepsis is unclear.

### 6.2 Influencing factors of PD-1 signaling pathway

The PD-1 signaling pathway, which plays an immunomodulatory role in sepsis, is regulated by a variety of factors. ​The pathogenic components such as lipopolysaccharide (LPS) (43) and the secreted toxins such as Streptococcal Pyrogenic Exotoxin A (SPEA) (44) can enhance the expression of PD-L1 in immune cells. Tissue hypoxia and its consequent hyperlactemia are also significant factors. The expression of PD-L1 in monocytes of septic patients increases under the regulation of transcription factor hypoxia-inducible factor-1α (HIF1α) (27). In septic mice, blood lactic acid can increase the expression of PD-L1 in the kidney and increase kidney injury (45). Some hormones can also affect the expression of molecules in this pathway, for example, human ghrelin and growth hormone can reduce the expression of PD-1 and the number of Tregs in the spleen of sepsis rats (46), and Vitamin D related signaling pathways can affect the expression of PD-1 and other molecules (47). The molecular mechanisms of these known influences and their effects on the expression of PD-1 signaling pathways in different cells need further investigation. In addition, there may be additional potential influences that have not yet been identified.

### 6.3 PD-1 signaling pathway and exosomes

Exosomes are the extracellular vesicles (EVs) with a diameter of 40 to 160nm, which are formed by the lipid bilayer coated with DNA, RNA and proteins, and play an essential role in mediating intercellular signal transmission and influencing gene expression in recipient cells (48). Exosome PD-L1 from tumor cells can bind to PD-1 on the surface of T cells, inhibiting the activity of the latter and creating an immunosuppressive microenvironment for tumors (49). Removal of exosome PD-L1 can inhibit the growth of tumor cells and has a synergistic anti-tumor effect with PD-L1 antibody (50). EVs are involved in the formation and organ function impairment of sepsis (51), and β2 integrin and PD-L2 expression are significantly increased in exosomes of sepsis patients (52). However, whether expression of PD-1 pathway molecules in the exosome has a direct regulatory effect on immune cells associated with sepsis and the specific mechanism remains to be investigated.

### 6.4 Soluble PD-1 pathway molecules

Besides the usual membrane-binding form, PD-1 and PD-L1 are dissolved in the circulating serum called soluble PD-1/sPD-L1 (sPD-1/SPD-L1), including the exosome PD-L1 mentioned above. Human sPD-1 level is positively correlated with the expression of PD-1 in CD4+ or CD8+T cells and the severity of sepsis (53). Serum sPD-L1 has also been shown to be associated with sepsis severity and prognosis (54). As soluble PD-1 pathway molecules are easy to extract and detect, they have the potential to be used as risk and prognostic indicators of sepsis. However, large-scale prospective clinical trials are currently lacking.

### 6.5 Metabolomics studies of PD-1 pathway in sepsis

Metabolomics can investigate the pathogenesis of sepsis and evaluate biomarkers and prognosis by detecting and analyzing metabolites (55). Bu et al. used a non-targeted metabolomics approach to investigate differences in metabolites in sepsis patients with different prognoses and PD-1 expression groups. They identified three metabolites: PC (P-18:0/14:0), 2-ethyl-2-hydroxybutyric acid and aldehyde increased significantly in 7-day death group and PD-1 high expression group. And 2-ethyl-2-hydroxybutyric acid is positively correlated with IL-2 and lactic acid concentration (56). These three metabolites may be involved in the mechanism of regulation of sepsis by the PD-1 pathway, and are closely related to environmental factors such as IL-2 and lactate. However, due to the limited number of samples, this is only a preliminary conclusion and further research is needed.

### 6.6 Immunomodulatory therapy

The therapeutic idea of improving immune function, destroying immune tolerance and immune escape by blocking the PD-1 signaling pathway was originally derived in tumor biotherapy. Up to now, more than 1700 clinical trials have been successfully conducted on tumor immunomodulatory therapy, and such drugs have been widely used in clinics, confirming the effectiveness and safety of immunomodulatory therapy (57). However, as mentioned above, the application of immunomodulatory therapies to sepsis has not been extensively studied, and current animal experiments targeting the PD-1 signaling pathway have not achieved fully consistent results.

The timing of treatment may be a key factor. Unlike the chronic course and low heterogeneity of tumors, sepsis occurs and develops quite rapidly. The pathogenic mechanism of different pathogens, the immune response intensity of the host individual and the basic organ function vary considerably, and the overall immune status is also continually changing (58). As a result, different timing of interventions may lead to differences in overall outcomes. Professor von Knethen et al. (38) enhanced PD-L1 expression in septic mice receiving cecal-ligation and puncture (CLP) under 24 hours, which inhibited the function of CD8+T cells to secrete inflammatory cytokines. In the early stages of sepsis, the main lethal factor is direct organ damage caused by excessive inflammatory cytokines. This intervention suppresses the early inflammatory response and protects organ function, making it reasonable to expect an improved prognosis. On the other hand, immunoregulatory therapies targeting the PD-1 pathway focus on persistent immunosuppression and double infection in late-stage sepsis, which are not contradictory. Of course, more research is needed to see if the results of animal trials can be applied in clinical applications.

In addition, it is difficult to accurately distinguish “early” and “late” sepsis in clinical cases, and the timing of immunoregulatory treatment still needs to be guided by indicators that accurately reflect the immune status of the host. Besides sPD-1/sPD-L1 mentioned above, Th17/Treg ratio and neutrophil/lymphocyte ratio (NLR) can be used as indicators of immune status (3). And the emerging artificial intelligence and machine learning technologies in recent years also have great application potential to guide the immunoregulatory therapy of sepsis (59).

## 7 Conclusion

In summary, the PD-1 signaling pathway plays an essential regulatory role in immunosuppression in sepsis by affecting immune cell function. Immunomodulatory therapies targeting the PD-1 pathway in sepsis have great potential for applications, but there are still many fundamental and clinical issues to be further investigated.

## Author contributions

YY designed the review and SZ wrote the manuscript. All authors contributed to the article and approved the submitted version.
